# Policy entrepreneurs and structural influence in integrated community case management policymaking in Burkina Faso

**DOI:** 10.1093/heapol/czv044

**Published:** 2015-10-29

**Authors:** Jessica C Shearer

**Affiliations:** Monitoring and Evaluation, PATH, Seattle, WA, USA

**Keywords:** C-IMCI, integrated community case management, policy analysis, policy entrepreneur, social network analysis

## Abstract

Policy entrepreneurs are individuals who attempt to influence the policy process and its outcomes through their opportunistic or incremental actions. Their success in the policy-making process has been associated with the convergence of four factors: behavioural traits; institutional factors; network position and political capital. Policy entrepreneurs have received little study in low- and middle-income country policy research despite observations of individualized decision-making, informal institutions and the unequal distribution and exercise of power in policymaking. This article aims to identify whether policy entrepreneurs were present in the policy process around integrated community case management (iCCM) in Burkina Faso, whether they were successful in achieving policy change, and whether success or failure can be explained using existing policy entrepreneur frameworks from high-income polities. This mixed methods policy study collected data from in-depth qualitative interviews and social network surveys of actors involved in iCCM policymaking [known locally as C-integrated management of childhood illness (IMCI)]; data were analysed based on the framework categories. Interview data pointed to one key individual who played a significant role in the inclusion of pneumonia treatment into the country’s iCCM policy, an issue that had been a point of contention between government policy elites and development partners. Social network data confirmed that this actor was strategically located in the policy network to be able to reach the most other actors and to be able to control the flow of information. Although some development partner actors were as strategically located, none had the same level of authority or trust as was imbued by being a member of the government civil service. The entrepreneur’s mid-level rank in the health ministry may have encouraged him/her to invest political capital and take risks that would not have been feasible or attractive to a more senior actor. This study highlights the convergence of factors needed to be an entrepreneur, as well as the role of development partner actors in creating a facilitating environment.

Key Messages

- Policy entrepreneurs are effective when they have certain behavioural traits, institutional constraints, political capital and network position.
- Mixed methods research, including social network analysis, can identify policy entrepreneurs.
- Policy entrepreneurs played a significant role in the introduction of iCCM policy in Burkina Faso.
- This study found that a successful policy entrepreneur in the Ministry of Health was willing to invest political capital, could leverage his/her institutional affiliation and occupied a strategic network position.

## Introduction and background

In studying why policies change, researchers have highlighted the role of policy entrepreneurs. ‘Policy entrepreneurs’ are individuals in the policy process who advocate for a specific policy proposal, build coalitions or secure political action and are willing to invest their own resources as well as their social and political capital in hopes of a future payoff (Kingdon 2003; Mintrom and Norman 2009). Policy entrepreneurs gained their name because of their high degree of entrepreneurial flair during policymaking, including a propensity for leadership, persuasion, persistence and innovation (Kingdon 2003; Mintrom and Norman 2009). Policy entrepreneurs do not have to be in a formal position of authority; rather, they tend to occupy a strategic structural position in their social and professional networks (Christopoulos 2006; Lewis 2006; Mintrom and Norman 2009). In short, actors must not only want to advocate, they must occupy a subset of the political space that allows them to take political risks and facilitates the diffusion and adoption of their ideas.

Policy entrepreneurs were observed during drug policy change in Malawi, Uganda and Zambia (Hutchinson *et al.* 2011), but despite the relevance of the framework, there have been few additional applications to health policy in low-income countries. Policy entrepreneur theory is consistent with other comparative public policy explanations of why policies change in Africa, including the role of informal institutions (Helmke and Levitsky 2004; Bratton 2007) and ‘Big Man’ rule (Hyden 2006). The application of policy entrepreneurship concepts, incorporating network analysis, could be instrumental in identifying sources of power and their consequences on health policymaking in this context.

This study aims to identify whether policy entrepreneurs were present in the policy-making process for integrated community case management (iCCM) in Burkina Faso, to what extent their characteristics, actions and outcomes map onto existing policy entrepreneur frameworks applied to high-income polities (Christopoulos 2006; Mintrom and Norman 2009) and to what extent these entrepreneurs were successful in achieving their policy goals.

## Methods

### Theoretical framework

To study the factors that facilitate and constrain entrepreneurs, one must consider a range of micro- and macro-level factors. Adapted from Christopoulos (2006), a policy entrepreneurs’ effectiveness can be explained by the convergence of four domains: behavioural traits, institutional constraints, network position and political capital. Christopoulos (2006) suggests that all four domains are necessary for successful entrepreneurship. As compelling as it may be to dichotomize these domains as either behavioural or structural (in an attempt to look for levers), it is perhaps more useful to instead think of each as being expressed on a continuum that spans individual agency to structural constraint. In short, none of the domains are impossible to change; in none can change be guaranteed.

Christopoulos (2006) approaches this issue by differentiating between opportunistic and incremental entrepreneurs. Opportunistic actors are typical of those described during agenda setting; they move an issue onto the agenda using a combination of persistence, good negotiation skills and an awareness of how they can engineer the policy network to improve their relative position (Kingdon 2003; Christopoulos 2006). Incremental actors align more closely with theories of advocacy coalitions; entrepreneurs find themselves in a strategic network position and possess the foresight to use that position to their advantage. These actors typically cultivate relationships with decision-makers and authority figures to demonstrate trust-worthiness (Mintrom and Norman 2009). Policy issues that are highly technical seem to favour the incremental entrepreneurship of policy analysts and bureaucratic actors (Rabe 2004).

Behavioural traits refer to intrinsic traits of an individual, independent of their environment, networks or institutions. These include rhetorical ability, foresight, persistence and good negotiating skill (Kingdon 2003; Christopoulos 2006), as well as social acuity (Mintrom and Norman 2009). Effective behavioural traits are often necessary to amass political capital, strengthen network position and leverage or circumvent institutions; indeed the impact of positive behavioural traits is multiplied in a supportive institutional environment. Rabe (2004) found that policy entrepreneurs in the climate change policy domain in USA persisted over many years to build strategic coalitions and took advantage of windows of opportunity when they arose. Hutchinson *et al.* (2011) also found that policy entrepreneurs in Malawi, Uganda and Zambia actively linked key groups and used foresight to identify strategic points for change. Kingdon (2003) found that rhetorical ability alone was not sufficient; an actor had to be listened to, and others were more likely to listen if the actor was an expert, was able to speak for others or had decision-making authority. Hypothesis: entrepreneurs are more likely to be persuasive communicators, possess foresight and persistence and have good social acuity*.*

Institutional constraints refer to institutions in the expansive sense—formal and informal rules of the game, organizational structures and social and cultural norms—and how they constrain actor- and group-level opportunities for action (North 1990). Rules of the policy-making process determine who has a seat at the table, while cultural and social norms partly determine who will be trusted. In their study of policy development for cotramaxizole prophylaxis in Malawi, Uganda and Zambia, Hutchinson *et al.* (2011) found that successful policy entrepreneurs held senior policy positions in well-funded organizations, although Kingdon (2003) argued that seniority was poorly correlated with entrepreneurship, as senior leaders tend benefit more from maintaining the *status quo* than changing it (Valente 2012). In reviewing how policy entrepreneur theories relate to new institutionalism, Mintrom and Norman (2009) stressed the importance of having an ‘insider’ sensibility, or a deep understanding of the social and cultural institutions, to be a successful entrepreneur. This type of social acuity is partly behavioural, and partly the effect of strategic use of network position (Krackhardt 1990; Mintrom 1998).

Institutional rules can be altered, which seems a strategic pursuit fitting of an opportunistic entrepreneur. Changes in institutions will ultimately change network structure by determining who participates and how resources are distributed, which in turn influences access to and distribution of political capital. Hypothesis: Entrepreneurs are more likely to be based in health ministries or have an insider perspective; they will not necessarily occupy senior leadership roles.

Network position refers to an actor’s specific location in their network of professional or social relationships and how that position affects their relative power and influence. Although formal institutional position is generally clear, network position requires empirical network mapping and the application of social network analysis algorithms to measure network actors’ connectedness (Knoke and Yang 2008). In policy sciences, network mapping has been used to describe the structure of influence in health policy networks (Lewis 2006; Oliver 2013), and to help explain policy change (Sandstrom and Carlsson 2008; Lubell *et al.* 2012). In the African context, network mapping may help to measure what Hyden (2006) calls the ‘social logic that is not captured by conventional western models [of policy change]’ (p. 264).

Less of the policy network literature has dealt explicitly with power, but the theoretical and empirical relationships between network position and power exist (Krackhardt 1990), and networks offer a valuable lens for exploring power in global health policy (Hanefeld 2015). If we consider power to be the ability to make others do things, then a powerful network position would be that which is highly connected, as measured by degree centrality (i.e. the count of an actor’s ties to others). Political scientists have persuasively argued for the use of a different type of centrality to measure actors’ structural influence in policy networks. Betweenness centrality measures the number of pairs of actors the focal actor sits between, indicating their theoretical ability to control information flow, act as a broker between otherwise unconnected actors and be exposed to new information (Freeman 1979; Krackhardt 1990). Power derived from this network position is essential for policy entrepreneurs, as entrepreneurs are typically able to access ideas from external networks, and broker otherwise unconnected actors within their network (Burt 2004; Mintrom and Norman 2009; Valente 2012). Although incremental entrepreneurs may find themselves in strategic network positions, opportunistic actors may try to reshape the network by changing institutional rules or through old-fashioned political savvy (Kingdon 2003). Hypothesis: Entrepreneurs are likely well-connected in the traditional sense (i.e. high degree centrality) but to also occupy a brokerage position in the network (i.e. high betweenness centrality).

The political capital dimension captures an actor’s stock of political capital and their willingness to invest it. ‘Political capital’ is adapted from Pierre Bourdieu’s (1989) writing on theories of capital and has been adopted by sociologists, network scholars and political scientists to describe the resources or benefits conferred by a political actor’s social structure (Coleman 1988; Bourdieu 1989; Lin 1999; Burt 2000; see Shiffman 2014; Hanefeld 2015) for applications to global health policy. Policy entrepreneurs are posited to have greater access to political capital, as well as a higher willingness to invest it in hope of some future return. An actor’s access to political capital is closely related to network structure, where their position in the network and the overall network structure will partly determine the distribution of capital and power throughout the network (Krackhardt 1990; Burt 2000; Sørensen and Torfing 2003). Opportunistic actors may seek to improve their network position in an attempt to access political capital whereas incremental entrepreneurs tend to find themselves in a desirable position (Christopoulos 2006). Access to political capital has also been related to behavioural traits and formal position of authority (Kingdon 2003), and, in the case of many Sub-Saharan African polities, to local norms where political capital is derived from honour, prestige or recognition (Hyden 2006).

What sets policy entrepreneurs apart from other actors with high stock of political capital is the willingness of entrepreneurs to invest it (Kingdon 2003)—a political opportunity function that varies even amongst entrepreneurs. Christopoulos (2006) found that actors with lower baseline levels of political capital were more likely to engage in high-risk opportunistic actions but became more conservative as their stock increased. Investment risk is mitigated by certain network structures, where density of trust reduces the transaction costs of asking advice or favours from others (Hawe and Shiell 2000). In assessing potential benefits of investment, entrepreneurs are likely to consider the institutional constraints around them. For example, Kingdon (2003) argued that policy entrepreneurs’ investments are driven largely by self-interest—protection of turf and resources, facilitating promotion, etc.—and it follows that if these benefits are perceived to be unavailable or unattainable due to institutional factors (i.e. rules dictating a fixed-term appointment), an actor will not invest. Hypothesis: entrepreneurs are more likely to have access to political capital by virtue of their network position, and have more to gain and less to lose from investing their capital.

### Study setting

This study was carried out in Burkina Faso, a low-income francophone country in West Africa. Burkina Faso was selected as a study country for a larger research project, which aimed to compare networks of policy actors around three policy domains in the Ministry of Health (MoH): the community case management of childhood illnesses (iCCM); the home management of malaria and the removal of user fees for antiretroviral treatment for HIV. Each of these policy decisions were made in 2008; this article focuses on decision making that led to the introduction of the iCCM programme in two administrative regions in Burkina Faso. The larger study involved a collection of qualitative and network data to describe and analyse policy-making processes and the effect of policy networks on policy outcomes for the three issues.

### Data collection methods

A document review was undertaken to establish an understanding of the iCCM policy process and its actors. Documents were sought that pertained to child health, community health and iCCM from the following libraries, databases and websites: the Burkina Faso MoH; Division of Family Health library; UNICEF Burkina library; WHO Burkina library; Department of Studies and Planning library; Google; PubMed; lefaso.net (newspaper) and lepays.com (newspaper). Search terms focused on ‘C- integrated management of childhood illness (IMCI)’ (‘Prise en charge integree des maladies de l’enfant, communautaire’ in French), the local name for the iCCM policy.

Interviews were sought with individuals who played a role in or who were knowledgeable about C-IMCI in Burkina Faso and comprised two components: an in-depth interview and a network survey. Social network analysis assumes that a network census is empirically measurable by asking actors to report their ties to others in the defined network. In this way, both respondents, and the data, are simultaneously identified through the same question, known as a ‘name-generator’. I first identified two focal actors based on the document review and then used the name generator to identify additional respondents, asking the question: ‘With whom did you interact during policymaking for iCCM?’ Actors were prompted to provide as many names as possible and these names were sought for interviews. Following other social network analysis (SNA) studies with policy actors (Lewis 2006), sampling continued until a new round elicited fewer new names than the preceding round, suggesting a full census of the network. Additional interviews were sought with actors who were not directly involved in the process, but who perhaps should have been, or who had an important perspective or stake in the issue. These reported interaction ties form the network data for this study.

The in-depth interview was semi-structured and based on a pre-established question guide touching on themes related to institutions, interests/actors and ideas in policy change, as well as the role of networks and innovation during the policy-making process. The survey elicited basic demographic and job-related information in addition to the network ties described earlier.

Interviews lasted on average for 45 min and were conducted in French. Interviews were audio recorded and notes were taken. Signed consent was received from respondents prior to beginning interviews, and ethical approval for the study was received from the McMaster University Faculty of Health Sciences Research Ethics Board and the Burkina Faso MoH’s Council National d’Ethique de la recherche en santé.

### Data analysis methods

In-depth interview recordings were transcribed in French and analysed in English. NVivo software was used to manage and code interview data. Coding was based on a pre-established codebook of the study themes and theoretical frameworks guiding this specific study as well as the related multi-country study on the adoption of iCCM policy (see other studies in this supplement). Efforts were made during analysis to identify emergent codes as well as negative data.

Data from the network survey were entered in a matrix of actors where cell values were coded as 1 or 0 depending on whether an interaction tie was reported or not between a pair of actors. Ties were coded as directed, so that ‘in-degree’ counts the number of nominations a given actor received from others, and ‘out-degree’ counts the number of other actors a given actor nominated during the survey. This distinction was made to identify actors who were reported by others as being most visible, as opposed to allowing an actor’s nominations of others to inflate their degree score. Social network data were analysed using the ‘Statnet’ suite of packages in R (Handcock *et al.* 2008) using existing SNA algorithms, including network size, density, centralization, degree centrality and betweenness centrality.

## Results

**Figure 1.**
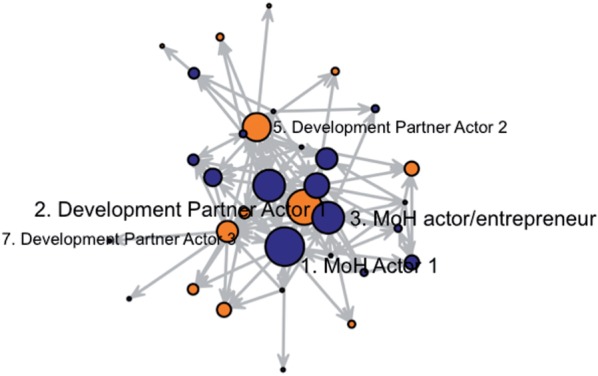
Interaction network, nodes sized and ranked by in-degree centrality. Circles represent actors in the network; Lines between nodes represent reported interactions, with arrow specifying direction of reported relationship; Blue colour indicates government actors, orange colour indicates development partners; Node size represents in-degree centrality scaled by a factor of 0.25 for optimal visualization; node coordinates are fixed to enable comparison between Figures 1 and 2

**Figure 2.**
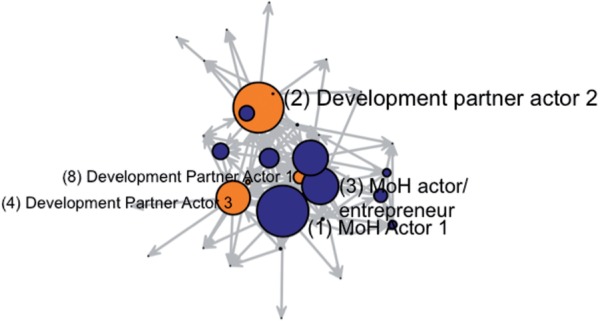
Interaction network, nodes sized and ranked by betweenness centrality. Circles represent actors in the network; Lines between nodes represent reported interactions, with arrow specifying direction of reported relationship; Blue colour indicates government actors, orange colour indicates development partners; Node size represents betweenness centrality scaled by a factor of 0.03 for optimal visualization; node coordinates are fixed to enable comparison between Figures 1 and 2

Thirty-four unique actors were identified through initial document review and the network survey as having participated in the C-IMCI policy network. Twenty-two were reached for interview, and 21 completed network surveys. Descriptive network data are described in Table 1, and network graphs are illustrated in Figures 1 and 2. Table 2 provides the in-degree and betweenness centrality scores for the top 10 nominated actors in this network, ranked in order of their in-degree (i.e. nominations received during the survey). These data will be discussed in greater detail alongside the qualitative results, later.

**Table 1.** Descriptive network statistics

Statistic	*N* or Mean (SD)
Nodes	34
Ties	614
Density	0.11
Centralization (in-degree)	0.23
Mean in-degree centrality	3.50 (3.00)
Mean betweenness centrality	22.6 (36.9)

**Table 2.** Centrality scores of 10 most central policy actors (ranked by in-degree)

Policy actor and organization	In-degree	Betweenness (rank)
1. MoH actor 1	11	126.949351 (1)
2. Development partner actor 1	10	30.83658
3. MoH actor/entrepreneur	9	90.37868 (3)
4. MoH actor 2	9	47.380303
5. Development partner actor 2	8	124.066017 (2)
6. MoH actor 3	7	86.902381 (4)
7. Development partner actor 3	6	83.246104 (5)
8. MoH actor 4	6	0
9. MoH actor 5	5	39.766667
10. MoH actor 6	4	20.283333

In an effort to protect confidentiality in the reporting of the study results, broad job categories are employed (i.e. MoH actor, development partner actor) and gendered pronouns are avoided in favour of ‘s/he’ and the singular ‘they’.

### History of C-IMCI in Burkina Faso

As in other countries, iCCM—called C-IMCI in Burkina Faso—first emerged on the policy agenda as part of discussions surrounding IMCI policy in the late 1990s. In its original form, C-IMCI was the education- and referral-based component of IMCI with no intention to allow s to treat childhood conditions at the community level. While the community-based treatment of childhood malaria, diarrhoea and malnutrition had been allowed and supported by the government for years with varying levels of implementation, MoH policy elites were cohesive in their opposition to the use of antibiotics by community health workers (CHWs), and thus the community-based treatment of pneumonia. Efforts by development partners to share positive experiences of other countries had been unsuccessful, with policy elites firm in their normative beliefs that CHWs’ low levels of literacy and training would compromise safety and effectiveness.

Well, it must be said that it was a bit controversial. We were a bit, truly skeptical, one would say… but this was also the position that the Ministry gave. To give some background, I know there was a study that the WHO met regarding, on the community-based management of acute respiratory infections. They presented it but at this time the Ministry truly rejected it, we were apprehensive, we said at the very least we need more evidence… (MoH; 888)

A visit by a Senegalese project in 2005, sponsored by US Agency for International Development (USAID), aimed to convince MoH decision makers that community-based management of pneumonia, alongside the other iCCM components, was feasible but these decision makers remained unconvinced.

Yes, we discussed it, I believe during 2004 or 2005, but we did not fall into agreement, as I said, we were not in agreement with putting antibiotics into the hands of CHWs in light of their capacity. (MoH; 132)

Sabatier and Weible (2007) point out the inherent difficulty in changing normative beliefs of policy actors, and that divergent beliefs between policy coalitions can lead to extended gridlock and conflict between groups, and yet increasing density of ties within groups.

### Opportunistic entrepreneurship by funders and development partners

The first major policy window opened in 2008 with the offer of grant funding to implement an expanded C-IMCI programme, including the treatment of all childhood conditions, in two health regions. The funders, The Partnership for Maternal, Newborn and Child Health and the Bill and Melinda Gates Foundation introduced the grant as a means of accelerating progress towards the UN Millennium Development Goals in Burkina Faso and used their financial and normative influence to strategically define the policy problem and to boost its importance on the decision-making agenda (Kingdon 2003). The grant had emerged from global discussions between funders and UN agencies, and indeed, UN agencies were involved from the start as supportive actors. The funders were not involved in day-to-day policy and proposal development for Burkina Faso’s grant submission but mandated UNICEF’s co-leadership in the grant-writing process alongside the government, thus ensuring representation of their opinions. The introduction of those institutional rules ultimately restructured the policy network to favour the funders’ interests, by the inclusion of UNICEF at the table. Meanwhile, UNICEF’s efforts to frequently communicate information and research evidence to government actors may have increased UNICEF’s network centrality and their control of information flow.

The overall composition in the network is illustrated in Figures 1 and 2, where a highly connected network core is visible, with fewer actors on the margins. The network seems to be somewhat centralized around Development Partner 1, who was mentioned frequently in relation to the technical work, as a co-leader in the process and as a source of research evidence, had the second highest in-degree centrality (9; Table 2). However, this actor nearly disappears on Figure 2, representing a much lower betweenness centrality score (30.8). Despite being highly connected and central in the traditional network sense, Development Partner 1 was not in a position to act as a broker between other actors and span diverse parts of the network. While this actor was willing to invest political capital in efforts to persuade colleagues, s/he may not have been adequately positioned within the network to succeed. Other development partners had higher betweenness centrality than Development Partner Actor 1, namely actors in development partner offices with higher job ranks (Development Partner Actor 2, betweenness = 124.1 and Development Partner Actor 3, betweenness = 83.2), reflecting their formal brokerage responsibilities as senior country-office leaders, as well as their seniority and experience in the health sector. But typical of actors with higher job rank, their lower in-degree scores suggest that they were not as active in direct engagements with many others.

### Cohesive network of MoH leadership

UNICEF and UN colleagues initially faced deeply held opposition from the cohesive network core of MoH policy elites on the issue of pneumonia management. Respondents recalled meeting at least twice a month, and sometimes for days on end, to complete the grant application. These meetings were led by the MoH focal person (MoH Actor 1 in Table 2) who was the official liaison between MoH and development partners, met monthly with the Secretary of Health, and was, by all accounts, a strong actor in the MoH hierarchy. Respondents described him/her as ‘powerful’ (Development partner actor; 393) and s/he was considered an effective communicator. The network data are consistent with this actor’s formal role and behavioural traits: they had the most nominations from others (11) and sat on the greatest number of paths between others (126.9). Despite this, there was no evidence that this actor invested political capital or acted entrepreneurially on the policy issue.

Persistent advocacy and communication by development partners to persuade the MoH to include CCM of pneumonia was unsuccessful; the government’s first grant submission in May 2008 included C-IMCI for malaria, diarrhoea and malnutrition but not pneumonia. Despite the funders’ ability to opportunistically structure the process and the network in ways that supported their policy goals, their efforts at entrepreneurship could not overcome the deeply held normative beliefs of MoH elites related to pneumonia treatment (Sabatier and Weible 2007).

### Incremental entrepreneurship by an MoH actor

The funders replied that the submitted proposal—without pneumonia—would not be accepted. This led to temporary gridlock between sides of the issue with no clear ‘out’ for either group. A second policy window opened during this period, when UNICEF invited MoH stakeholders from this network to attend an August 2008 meeting of international iCCM experiences in Madagascar. According to multiple respondents, one MoH actor returned from the meeting convinced of the effectiveness and feasibility of community-based pneumonia management and communicated it to others:

Yes, I was convinced. I was convinced that like in other countries where community health workers, possessing a certain level, regularly trained, supervised, could correctly manage pneumonia among infants under five years of age. Voila, the conclusion that I drew when I returned…

Yes, I, for example, I presented, at each time I presented the experience that I observed in Madagascar. With the experiences of Senegal, of Malawi, experiences of Rwanda… all those countries. In any case I gave presentations and that helped people, to convince people… (MoH; 226)

This actor’s formal role and responsibilities in the C-IMCI grant process can be considered a facilitating ‘rule of the game’ (i.e. institutional variable). Exposure to new networks and new information in Madagascar increased this actor’s stock of political capital and their effective and persistent communication, social acuity and trustworthiness enabled them to advise and inform other actors.

Each time I came (to a meeting) I encountered [him/her]. It’s [s/he] who… [s/he] went to certain countries to see a bit how it could work, and so, there, it was [s/he] who returned… It was [him/her] who returned and who convinced us that this [C-IMCI] works. (MoH; 883)

The MoH entrepreneur actor occupied a strategic network position (Row 2, Table 2). His/her in-degree score (9) indicated a high level of recognition in the network and the betweennness score (90.4) suggests broad brokerage and spanning ability in this network. Institutionally, this individual was in the MoH but not so high up that s/he was bound by the embedded norms that may have constrained his/her superiors. Being lower down in the MoH hierarchy may have increased his/her willingness to invest political capital, allowing risk taking and innovation where his/her supervisors would have been unable to do so. As noted by Mintrom and Norman (2009), ‘Policy entrepreneurs must be able to understand the workings of a given context without becoming so acculturated to it that they lose their critical perspective and their motivation to promote change’. Although development partners also continued to advocate strongly for policy change during this time, many respondents recognized his/her efforts as the key factor influencing the government’s decision to resubmit a proposal that included C-IMCI of pneumonia to be implemented on a pilot basis (Direction de la Sante de la Famille 2008). Table 3 summarizes the qualitative and network results for these actors.

**Table 3.** Policy entrepreneur domains observed for select policy actors

Domain	Definition	MoH actor 1	Development partner actor 1	MoH entrepreneur	Development partner actors 2 and 3
1. Behavioural traits	Intrinsic traits of an individual, including rhetorical ability, foresight, persistence and good negotiating skill.	Demonstrated leadership traits, ‘powerful’.	Persistent, good communicator.	Persistent, good communicator.	Persistent, good negotiators.
2. Institutional constraints	Formal and informal rules of the game, organizational structures and social and cultural norms.	Formal position as MoH focal point ensured access and credibility. Trusted by MoH colleagues.	Grant rules ensured participation in policy development.	Formal position as MoH participant ensured ability to exert influence. Trusted by MoH colleagues.	Grant rules ensured participation in policy development.
3. Network position	An actor’s specific location in their network of professional or social relationships, which can be measured empirically.	Formal role as leader of process ensured highest levels of in-degree and betweenness centrality.	Mandated role in decision-making improved relative network position; frequent efforts to communicate information and research evidence led to high in-degree centrality. Did not have equally high betweenness centrality.	Formal MoH role in process ensured relatively good network position. Persistent communication of lessons learned likely improved network position.	Mandated role in decision-making improved relative network position. Influence by virtue of formal role ensured good strategic network position.
4. Political capital	An actor’s access to and stock of political capital—resources, information or legitimacy conferred by social structure—and how willing they are to invest it.	High levels of political capital through formal position, but few incentives to risk it.	Access to financial resources and information ensures high level of political capital. Invested political capital in effort to persuade others.	Internal network position ensures access to political capital; exposure to new information and external networks at regional meeting increase stock. Willing to invest political capital as the risk of going against superiors’ normative beliefs balanced by reward of gaining favour with larger regional networks, and development partners.	Access to financial resources and information ensures high level of political capital. Potentially invested some political capital but not to same extent as other actors.

## Discussion

These results suggest that a policy entrepreneur participated in policymaking in ways that may have facilitated policy change. While multiple actors acted strategically to attain their policy goals, one entrepreneur seemed to be more successful. His/her success can be explained by the application of existing policy entrepreneur frameworks and network analysis; namely that entrepreneurial success requires a convergence of individual attributes and structural factors.

The contrast between the focal MoH actor and his or her MoH superior (MoH Actor 1) offers interesting insights into the role and limits of political capital as well as the contrast between ‘power’ in the formal, institutional sense and ‘power’ in the form of political capital. MoH Actor 1’s senior role and influential network position did not translate into entrepreneurship. His/her seniority may have limited incentives for risk-taking and entrepreneurship. Similar findings have been observed in high-income countries, where high-job level has been associated with reduced motivation to promote change (Mintrom and Norman 2009), as leaders tend to benefit from the status quo and thus have little incentive to invest their political capital in changing it (Valente 2012). Beyond this risk-benefit calculation, ideas and normative beliefs may have also played a role. Being embedded in the network core of policy elites may have increased this actor’s exposure to firmly held normative beliefs in opposition to pneumonia, and encouraged their congruence with those beliefs (Sabatier and Weible 2007).

The successful entrepreneur in this case occupied a network position that was highly strategic and yet was not senior or influential in the formal, organizational sense, consistent with the hypotheses. Being within the Ministry ensured credibility and access to others, while being any higher in seniority may have reduced incentives for innovation and risk-taking. This actor’s decision to invest political capital may have involved a conscious or sub-conscious calculation comparing potential benefits (i.e. recognition and visibility amongst colleagues) with potential risks (i.e. sanctions by managers). When s/he decided to act entrepreneurially, institutional constraints ensured that s/he had a seat at the table while cultural norms ensured that fellow decision-makers trusted him/her.

Funders and development partners had many entrepreneurial attributes and worked opportunistically to improve others, such as procedural rules and network position—a successful example of the investment of political capital in and of itself. Frequent dissemination of information and evidence further centralized UNICEF’s position in the network, and they were instrumental in bringing MoH actors, one of whom became significant in this narrative, to the evidence meeting in Madagascar. It is not clear whether their choice of attendees was strategic, although interview data from global-level iCCM policy actors suggests that these actors recognized the importance of sending the ‘right’ MoH stakeholders to the Madagascar meeting (Dalglish *et al.* 2015, submitted as part of this supplement). However, social network data demonstrated that while some development partner actors had many connections, and others had strategic positions, no actor was able to combine the two to be both as well connected and as strategically placed as the MoH entrepreneur; nor did they benefit from being inside the Ministry. Qualitative data suggested that the opinions of development partner actors may not have been as salient to government decision-makers, nor were they as trusted, as those of MoH actors, and particularly the entrepreneur in question. These findings are consistent with other settings, including in Malawi, where government technocrats are able to use their connections within the policy network, their legitimacy as government workers and their technical expertise to convince decision-makers (Rabe 2004; Hutchinson *et al.* 2011). The fact that iCCM is a relatively technical issue may also have facilitated the action of incremental insiders as opposed to opportunistic outsiders (Christopoulos 2006).

Finally, it is compelling to compare Development Partner 1’s degree and betweenness centrality in relation to their role in the policy process. Without network mapping, those wishing to disseminate information or ideas through this network would likely target that actor (as was actually the case for this policy process), noting that s/he communicated frequently with many other actors. Such an approach would ignore the particularity of social networks where the number of an actor’s connections does not translate into ability—according to network position—to reach the most in the fewest steps and to control information flow. Efforts to disseminate information or evidence for health policymaking would benefit from understanding the structure of social networks (Shearer *et al.* 2014; Yousefi-Nooraie *et al.* 2014).

Overall, the policy entrepreneur framework seems appropriate and applicable to policy change in low-income settings, and was generally able to explain variance in entrepreneurial behaviour and success rates across actors. The framework, which was adopted from the study of policy entrepreneurs in high-income countries (Christopoulos 2006), covered many of the particularities of policymaking in low- and middle-income countries and may be a more theorized way of explaining frequent observations of personalized decision-making, particularly in the African context (Gilson *et al.* 2003; Hyden 2006). Allowing a broad interpretation of the institutions category was necessary for a country where informal rules of the game are as significant as formal ones (Helmke and Levitsky 2004) and where sociological institutions can help to understand the role of social norms, trust and legitimacy in the study of policy change. Similarly, formal network mapping added value in terms of understanding players and power in a context where policy is frequently made by a diverse range of actors whose influence is derived less from formal position than from access to political and social capital. A core feature of the entrepreneur’s story was his/her exposure to other countries’ experiences through participation in regional and international networks at the behest of development partners. Because this was a case study of a single policy issue, it is difficult to pinpoint the effect of issue characteristics. These findings were consistent with hypotheses and previous research that government technocrats are more visible on technical policy issues (Rabe 2004; Moat *et al.* 2013).

## Limitations and future research

This article measured and reported interactions between policy actors; ties measuring perceived influence, or power, were not measured during network data collection as they have been in other studies of policymaker influence (Lewis 2006). Some actors were not reached for interviews or network surveys; the missing data is of greater concern for the network analysis, where missing ties could result in an underestimate of actors’ in-degree centrality. The decision to collect a cross-sectional snapshot of the overall network limits the ability to infer temporality or causality, and masks potential shifts in power over the duration of the policy-making process. While the interview question guide captured a detailed picture of actors, their alliances, and influence, it did not ask questions specific to ‘entrepreneurship.’ Future research should employ longitudinal data collection and more specific interview questions to test causal hypotheses related to the relationship between network position, opportunistic actions and policy outputs.

These findings around the role of policy entrepreneurs in health policy processes in low- and middle-income countries are somewhat unique and future research is needed to confirm these findings in other settings and for other policy issues. The highly technocratic nature of iCCM may be more amenable to policy entrepreneurship of someone in the MoH than would be a highly politicized policy issue with greater civil society involvement. Additional cases should also be sought to confirm or disprove the hypothesis that development partners and other external actors do not have the right mix of supportive institutional constraints and network position to achieve success as entrepreneurs, or alternately that they operate as entrepreneurs through embedded local actors. As in many studies of politics and policymaking in low-income countries, the specific and complex role of funders and development partners adds an extra layer to theory and empirical research borrowed from other settings.

## Conclusion

This analysis demonstrates the role of policy entrepreneurs in iCCM policy adoption in Burkina Faso, combining qualitative data describing actors’ roles in policy change with data on their network positions. While funders and development partners acted opportunistically, particularly during the agenda-setting phase, an actor within the ministry leveraged behavioural and structural attributes to successfully overcome gridlock and achieve policy change. This theoretical lens offers compelling insights as part of a supplement on iCCM policy change in six countries.

## References

[czv044-B1] Bourdieu P 1989 Social space and symbolic power. Sociological Theory 7: 14–25.

[czv044-B2] Bratton M 2007 Formal versus informal institutions in Africa. Journal of Democracy 18: 96–110.

[czv044-B3] Burt R 2004 Structural holes and good ideas. American Journal of Sociology 110: 349–99.

[czv044-B4] Burt RS 2000 The network structure of social capital. Research in Organizational Behavior 22: 345–423.

[czv044-B5] Christopoulos DC 2006 Relational attributes of political entrepreneurs: a network perspective. Journal of European Public Policy 13: 757.

[czv044-B6] Coleman JS 1988 Social capital in the creation of human capital. American Journal of Sociology 94: S95–120.

[czv044-B42] Dalglish SL, George A, Shearer JC, Bennett S. 2015. Epistemic communities in global health and the development of child survival policy: a case study of iCCM. *Health Policy and Planning*. in press 10.1093/heapol/czv043 26516146

[czv044-B7] Direction de la Sante de la Famille. 2008 Requete du Burkina Faso aupres du partenariat mondial pour la sante de la mere, du nouveau-ne et de l'enfant. Ouagadougou: Government of Burkina Faso.

[czv044-B8] Freeman LC 1979 Centrality in social networks conceptual clarification. Social Networks 1: 215.

[czv044-B9] Gilson L Doherty J Lake S 2003 The SAZA study: implementing health financing reform in South Africa and Zambia. Health Policy and Planning 18: 31–46. 12582106 10.1093/heapol/18.1.31

[czv044-B11] Handcock MS Hunter DR Butts CT Goodreau SM Morris M 2008 statnet: software tools for the representation, visualization, analysis and simulation of network data. Journal of Statistical Software 24: 1548–7660. 10.18637/jss.v024.i01 PMC2447931 18618019

[czv044-B12] Hanefeld J Walt G 2015 Knowledge and networks – key sources of power in global health. International Journal of Health Policy and Management 4: 119–121. 25674577 10.15171/ijhpm.2015.25 PMC4322625

[czv044-B13] Hawe P Shiell A 2000 Social capital and health promotion: a review. Social Science & Medicine 51: 871–85. 10972431 10.1016/s0277-9536(00)00067-8

[czv044-B14] Helmke G Levitsky S 2004 Informal institutions and comparative politics: a research agenda. Perspectives on Politics 2: 725.

[czv044-B15] Hutchinson E Parkhurst J Phiri S 2011 National policy development for cotrimoxazole prophylaxis in Malawi, Uganda and Zambia: the relationship between context, evidence and links. Health Research Policy and Systems 9(Suppl 1): S6. 21679387 10.1186/1478-4505-9-S1-S6 PMC3121137

[czv044-B16] Hyden G 2006 African Politics in Comparative Perspective. Cambridge: Cambridge University Press.

[czv044-B17] Kingdon JW 2003 Agendas, Alternatives, and Public Policies*.* 2nd edn, New York: Longman.

[czv044-B18] Knoke D Yang S 2008 Social Network Analysis*.* 2 edn, Thousand Oaks, CA: SAGE Publications.

[czv044-B19] Krackhardt D 1990 Assessing the political landscape: structure, cognition, and power in organizations. Administrative Science Quarterly 35: 342–69.

[czv044-B20] Lewis JM 2006 Being around and knowing the players: networks of influence in health policy. Social Science & Medicine 62: 2125–36. 16289737 10.1016/j.socscimed.2005.10.004

[czv044-B21] Lin N 1999 Building a network theory of social capital. Connections 22: 28–51.

[czv044-B22] Lubell M Scholz J Berardo R Robins G 2012 Testing policy theory with statistical models of networks. Policy Studies Journal 40**:** 351–74.

[czv044-B24] Mintrom M 1998 Policy networks and innovation diffusion: the case of state education reforms. The Journal of Politics 60: 126.

[czv044-B25] Mintrom M Norman P 2009 Policy entrepreneurship and policy change. Policy Studies Journal 37: 649–67.

[czv044-B26] Moat KA Lavis JN Abelson J 2013 How contexts and issues influence the use of policy-relevant research syntheses: a critical interpretive synthesis. The Milbank Quarterly 91: 604–48. 24028700 10.1111/1468-0009.12026 PMC3790526

[czv044-B27] North DC 1990 A transaction cost theory of politics. Journal of Theoretical Politics 2: 355.

[czv044-B29] Rabe B 2004 Statehouse and Greenhouse: the Stealth Politics of American Climate Change Policy*.* Washington, D.C.: Brookings Institution Press.

[czv044-B30] Sabatier PA Weible C 2007 The advocacy coalition framework: innovations and clarifications. In: Sabatier PA (ed). Theories of the Policy Process. 2nd edn, Boulder, Colorado: Westview Press.

[czv044-B31] Sandstrom A Carlsson L 2008 The performance of policy networks: the relation between network structure and network performance. Policy Studies Journal 36: 497–524.

[czv044-B32] Shearer JC Dion M Lavis JN 2014 Exchanging and using research evidence in health policy networks: a statistical network analysis. Implementation Science 9: 126 doi:10.1186/s13012-014-0126-8. 25358894 10.1186/s13012-014-0126-8 PMC4226903

[czv044-B34] Shiffman J 2014 Knowledge, moral claims and the exercise of power in global health. International Journal of Health Policy and Management 3**:** 297–9. 25396204 10.15171/ijhpm.2014.120 PMC4226618

[czv044-B35] Sørensen E Torfing J 2003 Network politics, political capital, and democracy. International Journal of Public Administration 26: 609–34.

[czv044-B37] Valente TW 2012 Network interventions. Science 337: 49–53. 22767921 10.1126/science.1217330

[czv044-B41] Yousefi-Nooraie R Dobbins M Marin A 2014 Social and organizational factors affecting implementation of evidence-informed practice in a public health department in Ontario: a network modelling approach. Implementation Science 9**:** 29. 24565228 10.1186/1748-5908-9-29 PMC3938902

